# *Strongyloides* questions—a research agenda for the future

**DOI:** 10.1098/rstb.2023.0004

**Published:** 2024-01-15

**Authors:** Reem Al-Jawabreh, Roy Anderson, Louise E. Atkinson, Jack Bickford-Smith, Richard S. Bradbury, Minka Breloer, Astra S. Bryant, Dora Buonfrate, Luke C. Cadd, Bethany Crooks, Michela Deiana, Warwick Grant, Elissa Hallem, Shannon M. Hedtke, Vicky Hunt, Virak Khieu, Taisei Kikuchi, Asuka Kounosu, Dominika Lastik, Lisette van Lieshout, Yuchen Liu, Henry J. McSorley, Paul McVeigh, Angela Mousley, Ben Murcott, William David Nevin, Eva Nosková, Elena Pomari, Kieran Reynolds, Kirstin Ross, Adrian Streit, Mona Suleiman, Natalia Tiberti, Mark Viney

**Affiliations:** ^1^ Life Sciences Department, University of Bath, Bath BA2 7AY, UK; ^2^ Department of Infectious Disease Epidemiology, Imperial College London, London SW7 2BX, UK; ^3^ Department of Infectious Diseases, Imperial College London, London SW7 2BX, UK; ^4^ School of Biological Sciences, Queen's University Belfast, Belfast BT9 5DL, UK; ^5^ The London School of Hygiene & Tropical Medicine, London WC1E 7HT, UK; ^6^ Federation University, Melbourne, Victoria 3806, Australia; ^7^ Bernhard Nocht Institute for Tropical Medicine, Hamburg 20359, Germany; ^8^ Department of Physiology and Biophysics, University of Washington, Seattle 98195, USA; ^9^ Department of Infectious Tropical Diseases and Microbiology, IRCCS Sacro Cuore Don Calabria Hospital, Verona 37024, Italy; ^10^ Department of Environment and Genetics, La Trobe University, Bundoora, Victoria 3083, Australia; ^11^ Department of Microbiology, Immunology, and Molecular Genetics, Molecular Biology Institute, University of California Los Angeles, Los Angeles 90095, USA; ^12^ National Centre for Parasitology, Entomology and Malaria Control, Cambodia Ministry of Health, Cambodia; ^13^ Department of Integrated Biosciences, Graduate School of Frontier Sciences, The University of Tokyo, Kashiwa 277-8652, Japan; ^14^ Division of Parasitology, Department of Infectious Diseases, Faculty of Medicine, University of Miyazaki, Miyazaki 889-1692, Japan; ^15^ Leiden University Center for Infectious Diseases, Leiden University Medical Center, 2300 RC Leiden, The Netherlands; ^16^ Department of Evolution, Ecology & Behaviour, University of Liverpool, Liverpool L69 7ZB, UK; ^17^ Division of Cell Signalling and Immunology, School of Life Sciences, University of Dundee, Dundee DD1 5EH, UK; ^18^ Department of Clinical Sciences, Liverpool School of Tropical Medicine, Liverpool L3 5QA, UK; ^19^ Department of Botany and Zoology, Faculty of Science, Masaryk University, 611 37 Brno, Czech Republic; ^20^ Institute of Vertebrate Biology, Czech Academy of Sciences, 603 65 Brno, Czech Republic; ^21^ Environmental Health, College of Science and Engineering, Flinders University, South Australia 5042, Australia; ^22^ Department of Integrative Evolutionary Biology, Max Planck Institute for Biology Tübingen, Tübingen 72076, Germany

**Keywords:** *Strongyloides*, questions, research

## Abstract

The *Strongyloides* genus of parasitic nematodes have a fascinating life cycle and biology, but are also important pathogens of people and a World Health Organization-defined neglected tropical disease. Here, a community of *Strongyloides* researchers have posed thirteen major questions about *Strongyloides* biology and infection that sets a *Strongyloides* research agenda for the future.

This article is part of the Theo Murphy meeting issue ‘*Strongyloides*: omics to worm-free populations’.

## 1. Introduction

**Figure 1.**
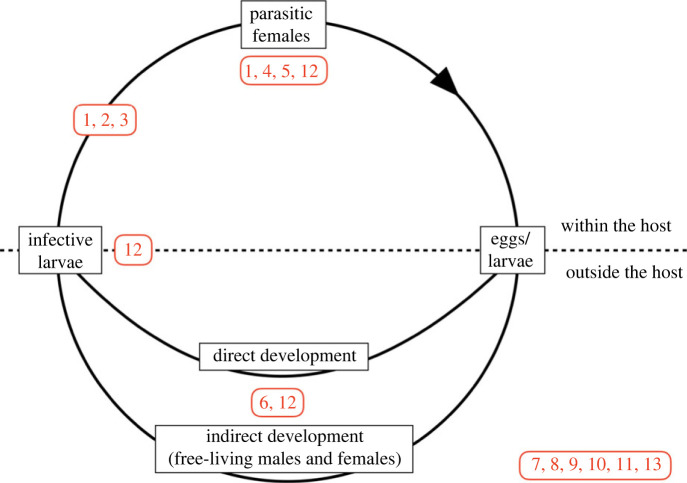
The life cycle of *Strongyloides*, with parasitic female worms inside hosts that produce eggs that pass out of the host, where larvae either develop (i) directly to infective larvae that infect a host and migrate to the host gut, or (ii) indirectly into free-living adult males and females, whose progeny develop into infective larvae, which then infect a host. *Strongyloides stercoralis*, the parasite of people, also undergoes internal autoinfection. Questions 1, 2, 3, 4, 5, 6 and 12 (shown in red) are about specific aspects of the *Strongyloides* life cycle and are shown at the point in the life cycle where they pertain; Questions 7, 8, 9, 10, 11 and 13 do not directly apply to the life cycle, and are shown separately.

*Strongyloides* is a genus of parasitic nematode that infects a wide variety of terrestrial vertebrates, including humans. *Strongyloides* is one of the soil-transmitted helminthiases, and so a WHO-defined Neglected Tropical Disease, and it is estimated that some 100–600 million people are infected with *Strongyloides* worldwide [1,2]. *Strongyloides* infection is also of some clinical veterinary relevance [3]. A recent ‘*Strongyloides*: omics to worm-free populations' meeting brought together a diverse, international group of people interested in *Strongyloides.* Directly after the meeting this community posed questions that they had about *Strongyloides*, in part inspired by similar question-setting by other research communities [4]. This resulted in 93 questions (see electronic supplementary material, table S1), of which 90 could be grouped into 13 main questions, divided into two main themes, Basic Biology and Immunology (8 main questions), and Human Infection and Disease (5 main questions). Many of these questions relate to aspects of the *Strongyloides* life cycle, shown in figure 1. In what follows, for each main question the current state of knowledge is used to give a context to the question and then state outstanding questions, so articulating a *Strongyloides* research agenda for the future. The questions considered here are clearly not an exhaustive list, and others will have additional, different questions.

## 2. Theme 1: basic biology and immunology

**Question 1. What is the biology of host infection and within-host physiological and behavioural adaptation?** (8 questions, electronic supplementary material, table S1)

The enduring fascination of parasites is that they live inside other animals, an environment that to us might seem hugely inhospitable. But, of course, they have evolved to live in these environments and have a suite of adaptations enabling them to do so. For *Strongyloides*, as for many other parasitic nematodes, there are a raft of questions about how this adaptation is actually achieved. *Strongyloides* infective third-stage larvae are developmentally arrested and non-feeding, and live in the soil. When these penetrate a host, host-derived signals allow them to detect that they are inside a host, which then initiates physiological and gene expression changes enabling larvae to exit developmental arrest and resume reproductive growth as the parasitism programme of development is initiated. There is a long history of studying what signals change the behaviour of parasitic nematode larvae, and these can be expanded to more fully understand the signals that *Strongyloides* uses to detect the within-host environment, beyond those already known [5–7]. An interesting question here is to what extent host-derived signals contribute to *Strongyloides*' host species-specificity (and see **Question 7**, below). Studying the signals that *Strongyloides* uses within hosts is more challenging because this would likely need to be addressed using *ex vivo* experiments. For the same reason, studying the within-host behavioural biology is also challenging, though the development of remote imaging of worms *in vivo* (possibly including transgenic worms that report signal reception) can develop this area. *Strongyloides ratti* parasitic females migrate through host intestinal tissue, laying eggs as they go, and disperse themselves along the gut. These phenomena and the underling behavioural biology are completely unexplored.

It is likely that the larval head sensory neurons are an important part of the sensory process of host infection, within-host migration, and other within-host biology [7,8]. The structure and function of nematode head neurons have been studied extensively in the free-living nematode *Caenorhabditis elegans*, and the structure of *Strongyloides stercoralis* head neurons have been determined, showing some anatomical similarity to those of *C. elegans* [5,9,10]. Moreover, functional studies have revealed that *Strongyloides*' head sensory neurons confer responses to the same sensory modalities as *C. elegans* [5,8,11–14], and at least some of the signal transduction pathways that mediate sensory responses are also conserved between *Strongyloides* and *C. elegans*. However, how transduction of these signals leads to changes in gene expression that result in within-host adapted biology remains unknown.

**Outstanding questions include:** what host signals do *Strongyloides* use to detect their within-host environment; how does the detection of these signals then lead to the initiation and then exposition of its parasitism programme; and what is the behavioural biology of *Strongyloides* within the host?

**Question 2. What is *Strongyloides*’ within-host migration route?** (3 questions, electronic supplementary material, table S1)

*Strongyloides*, in common with many parasitic nematodes, undergoes a within-host migration from the site of infection to the final within-host site. A phylogenetically controlled analysis of parasitic nematodes shows that such within-host migration allows developing worms to grow more (compared with those that do not migrate), with this greater adult size resulting in greater fecundity, and so likely greater comparative fitness [15]. For *Strongyloides*, the canonical route of migration has been thought to be from skin penetration, via the blood, lungs, trachea, to the intestinal tract, where parasitic adults establish [16]. But, working out the route of migration is less than straightforward. A substantial proportion of infective stages that infect a host never make it to the final within-host site. If larvae are found in various sites in a host shortly post-infection, are these larvae that are lost and will never get to the gut, or are they larvae *en route* to the gut? The logic of being able to confirm which larvae are *en* *route* to the gut was laid out by Tindal & Wilson, and for *S. ratti* in rats they and others convincingly showed that the naso-frontal region was a key part of the migration route of worms *en route* to the gut [17–19]. Investigating routes of within-host migration can be addressed by direct parasitological methods in some host species, though obviously not in human hosts. Knowing how, when, and where *Strongyloides* migrates in people is relevant not only to better understand pathology but also to diagnose infection.

**Outstanding questions include:** is there diversity in migration route (or routes) for different *Strongyloides* species or for the same species when in different hosts?

**Question 3. What is the diversity of routes of infection?** (6 questions, electronic supplementary material, table S1)

Successfully infecting a host is central to parasites’ evolutionary fitness, and for *Strongyloides* this occurs by infective larvae penetrating host skin, which has been studied in the laboratory. However, rather little of this is known in natural conditions, in no small part because of the substantial difficulty of finding *Strongyloides* infective larvae in nature or studying their infection of hosts. Understanding how *Strongyloides* infective larvae invade hosts under natural conditions is critical for an understanding of the basic biology of host infection and the epidemiological parameters that affect infection in host populations. Other routes of infection need to be considered, and there is some evidence of transmammary transmission in humans [20] and other animals, though inconsistently (e.g. [21–23]).

**Outstanding questions include:** what is the natural biology of *Strongyloides* infection and are there means of host infection other than skin penetration by infective larvae?

**Question 4. What is the role of certain pathways and of parasite-derived products?** (8 questions, electronic supplementary material, table S1)

Parasitic nematodes inside hosts release molecules and other products that can affect the host to the parasite's benefit. For example, helminth immunomodulation of hosts is now known to be due to host excreted/secreted products (ES) [24,25]. Other parasitic nematodes have been shown to release extracellular vesicles (EVs) that *in vitro* can affect host cells [26], and that contain proteins and non-coding RNAs that can directly influence host gene expression [27]. *Strongyloides* also produces ES [28–30] and EVs (V. Hunt 2023, personal communication), but understanding the nature of the contents of the EVs is an area of active work, as too is elucidating the function of the contents, as well as of the wider ES. The ES of *Strongyloides* parasitic females seem prodigious (shown empirically both by *in vitro* studies and by inference given the size of the gene families whose products are likely secreted inside hosts [29]), and one can envisage that these are involved in *Strongyloides*' residence in, and feeding on, hosts. *Strongyloides* parasitic females lie within, and migrate through, host tissue, and parasite-derived products likely play a role in regulating these processes. Genome sequencing projects have also identified various molecular pathways and other molecules in *Strongyloides*—such as the endocannabinoid pathway [31]—that may play a role in *Strongyloides* within-host biology.

Apart from understanding the normal role of parasite-derived products in hosts, the very fact that these factors are in the host could be exploited for diagnosis of infection or parasite control. Such approaches have not yet been developed for *Strongyloides*, and there remains the challenge of genus- or species-specific diagnoses (see **Question 10**).

**Outstanding questions include:** what molecules do *Strongyloides* release into their hosts, and what effects do these molecules have on the hosts and so on *Strongyloides* itself, and can these molecules be used to diagnose infection; what are the roles of a range of *Strongyloides* genetic pathways in its parasitic lifestyle?

**Question 5. What is the immunological relationship of *Strongyloides* and its host?** (3 questions, electronic supplementary material, table S1)

Many parasitic nematodes immunomodulate their hosts for their own benefit [24,25] and the mechanisms of how they do this remain under intense study. Several lines of evidence suggest that *Strongyloides* follows in this pattern; for example, *S. ratti* infection in mice expands T lymphocytes with regulatory function and induces the expression of regulatory receptors on effector T cells [32–35]. Importantly, abrogation of parasite immunomodulation through immunization-induced antibody blockade could be a viable vaccination strategy [36]. Most work on *Strongyloides* immunobiology in this area has been done in laboratory models, particularly with *S. ratti* and *Strongyloides venezuelensis*, but there is a comparative dearth of studies in people and livestock [37]. However, studies in humans show that there is protective immunity to *S. stercoralis* and that these immune responses include many general features of anti-helminth immune responses seen with other helminth infections [37,38].

**Outstanding questions include:** does *Strongyloides* immunomodulate its hosts (and if so how), and what is the functional effect of host anti-*Strongyloides* immune responses in natural human and animal infections; what is the extent of molecular communication from parasite to host, and host to parasite?

**Question 6. What is the *Strongyloides* life cycle, particularly the free-living generation, and does it vary among species?** (15 questions, electronic supplementary material, table S1)

*Strongyloides* has a complex and fascinating life cycle, and one that sets it apart from most other parasitic nematodes. Specifically, in *Strongyloides* external to the host there are direct (homogonic) and indirect (heterogonic) routes of development that ultimately result in infective larvae. Direct development is larval only, whereas indirect development involves free-living adult nematodes (figure 1). Details of this free-living development have only been studied in detail in a few laboratory-maintained species [39], though free-living adult stages have been described for a wider range of species. So, at best, our understanding of this phase of the *Strongyloides* life cycle is taxonomically restricted and thus the potential diversity of this life cycle in different species is unknown. *Strongyloides*’ nearest relative, *Parastrongyloides*, has multiple free-living adult generations [40], suggesting that the ability to have one or more free-living generations may vary among *Strongyloides* species, as has been shown at least once [41].

*Strongyloides*' direct development appears to be similar to the development of many other parasitic nematodes, but indirect development is quite distinct, which raises the question of why *Strongyloides* has been selected for this apparently rare life cycle. It is notable that among parasitic nematodes of vertebrates virtually all reproduce sexually, which contrasts with the wide range of sexual, hermaphroditic, and parthenogenetic reproduction among nematodes more widely. One possibility is that the parthenogenetic reproduction of *Strongyloides* parasitic females, and so the absence of sexual reproduction, has been compensated for by sexual reproduction in its indirect route of development.

*Strongyloides* larvae have a developmental choice between direct and indirect development, and some of the cues that affect this, such as environmental temperature, are known but how these developmental choices are molecularly specified remains to be discovered. Here, the fate of the offspring of parasitic and free-living females clearly differs. In the well-studied *S. ratti* system the progeny of free-living females always develop into infective larvae, whereas the progeny of parasitic females can be mixed, developing into infective larvae directly and into free-living adults. The control of these different fates is fascinating, and *Strongyloides* may be a very good system in which to investigate control of development fate in nematodes. The lifespan of the two adult female stages also differs, with the parasitic female living for a maximum of about a year, some eighty times the maximum 5 day lifespan of the free-living female [42]. The mechanistic basis of this difference is not understood [43], though the evolutionary theory of ageing would suggest that the parasitic phase of the life cycle is one with the lowest effective extrinsic mortality rate, either directly or because of the facultative nature of the free-living adult stage [42]. Analogies have been made between the direct and indirect development of *Strongyloides* and the dauer versus non-dauer choice of free-living nematodes (including *C. elegans*) [44], which have been explored for a range of parasitic nematodes including *Strongyloides*, though probably with little ultimate benefit [45,46]. However, recently it has been shown that Δ7-dafachronic acid specifies the development of *S. stercoralis* infective larvae, a mechanism that is directly analogous with *C. elegans* dauer larva development [47]. But there are also other levels of control that are not understood, for example how the sex of *Strongyloides* infective larvae is always female.

*Strongyloides stercoralis* undergoes autoinfection, where apparently precocious development occurs inside the host so that infective stages internally infect the host. How this is controlled and whether or not it is unique to *S. stercoralis* remain to be studied.

**Outstanding questions include:** what diversity (if any) is there in the free-living life cycle of different *Strongyloides* species, or genotypes within species; what are the cues that initiate direct or indirect development and how are these developmental pathways controlled, including the control of sex determination; what are the selection pressures acting on the free-living developmental route, and can they help explain autoinfection of *S. stercoralis*?

**Question 7. What is a *Strongyloides* species and what are species' host ranges?** (14 questions, electronic supplementary material, table S1)

There are currently more than 50 species of *Strongyloides* described, all based on morphological characters and/or the host from which they were derived. *Strongyloides* taxonomy is challenging: there are limited morphological characters, the adult parasitic female stages are not always available, and a number of putatively useful morphological characters can be altered by fixation and downstream processing [48]. More recently there have been molecular analyses of *Strongyloides*, commonly by sequence analysis of single loci, but with some whole genome sequencing too [49]. While molecular analyses may be a step forward in understanding *Strongyloides* populations and species, there is a substantial challenge in linking sequence data to *Strongyloides* species names. Often there is no explicit taxonomic basis for the species names that are attached to sequence data in databases, so these names should be treated with caution. Several *Strongyloides* species have been erected largely (or solely) on the basis of the host species in which the parasite was found. Almost analogously, recent molecular approaches often use host species to provide a *Strongyloides* species name to the parasite.

While the aim of taxonomy is to identify and distinguish species that are biologically meaningful, the very concept of a *Strongyloides* species may be unclear. Specifically, a species can be considered a group of inter-breeding individuals (though there are other species concepts), but in *Strongyloides* ‘interbreeding’ relies on the facultative free-living adult generation. Cleary these do exist, but outside of laboratory-maintained *Strongyloides* lines how often (if at all) they occur is unknown. In the absence of free-living sexual stages, *Strongyloides* will consist of lineages of parthenogenically reproducing genotypes, and in this scenario what is a *Strongyloides* species is a moot point.

Putting this together, for *Strongyloides* the whole species concept may be in doubt, but also there is no good taxonomic assignment of most species, and molecular identification and naming of species is even less clear.

Beyond the whole question of defining a *Strongyloides* species, the host range of *Strongyloides* species or genotypes is little known. Several *Strongyloides* species have been erected largely (or solely) on the basis of the host species in which the parasite was found. Almost analogously, recent molecular approaches often use host species to provide a *Strongyloides* species name to the parasite (e.g. [50,51], but see [52]). For *Strongyloides*, the common, implicit, assumption is that there is a one-to-one relationship between host species and *Strongyloides* species. This assumption is challenged when the same parasites are found in multiple host species—for example *S. stercoralis* in people, carnivores and great apes; *Strongyloides papillosus* in sheep and rabbits—and it is notable that these putative exceptions are in well-studied taxa such that one might wonder if they are rather the rule than the exception.

Studying the host range of parasites can be done by cross-infection studies, that is taking a parasite from host species X and attempting to infect host species Y. While possible for some hosts, it is clearly not possible or desirable for all hosts, and most certainly not for humans. Experimental models—*S. papillosus, S. ratti, S. stercoralis,* *S. venezuelensis—*have had their host species range examined experimentally in this way. This is not the place to review this literature, but the general pattern is that (i) some *Strongyloides* species can infect more than one host species, though with varying success, and (ii) severe immune suppression or modification is needed to break down host species barriers [53], suggesting that *Strongyloides*' host range is determined by a wider set of host and parasite physiological characters.

An alternative approach to understanding host range is population genetics; this is, identifying genetically defined *Strongyloides* populations and then asking how those align to host species. The complication here is that, given the potential asexual nature of the *Strongyloides* life cycle, the population genetic structure of populations may deviate from those expected under patterns of random sexual mating. Beyond the basic biological interest of understanding species’ niche breadth and parasite host range, the host range of *Strongyloides* species is of epidemiological importance for species infecting humans and livestock. Specifically, to control infection in a focal host species, one needs to know the source of infection, and whether that is just the focal host species or other host species.

To make progress our community needs to agree how to refer to *Strongyloides* genotypes, with a system that encompasses both molecular and non-molecular approaches. Given that it is now possible to whole genome sequence *Strongyloides* straight from the wild, there are great prospects for widely studying its population genomics, to bring an unrivalled understanding of the genetic diversity in *Strongyloides* and its association with hosts.

**Outstanding questions include:** can we apply the biological species concept to *Strongyloides* (which might only sexually reproduce occasionally, if at all) or should other species concepts be considered; how should we define and use species names (or, even, should we use species names) with *Strongyloides*; how are genetically related *Strongyloides* genotypes distributed among host populations and host species, and from this what are the sources of infection of humans and of livestock?

**Question 8. What laboratory methods do we need to improve, to better study *Strongyloides*?** (3 questions, electronic supplementary material, table S1)

This article presents outstanding questions in *Strongyloides* biology, and the development of new methods will help make progress with answering these questions. Understanding the within-host biology (**Questions 1** and **2**) will benefit from remote sensing or other tracking methods. Ultimately, understanding the role of molecular pathways and parasite-secreted molecules, and the control of *Strongyloides*' free-living generation (**Questions 4** and **6**) will require using reverse genetic approaches. *Strongyloides* is one of the few parasitic nematode species where there are established CRISPR-Cas9 and RNAi methods [54–56], though they remain technically challenging, especially for propagation through hosts. Study of *S. stercoralis* in humans will always be limited, though dogs and gerbils are available as experimental models (though see **Question 7**), and so the use of *in vitro* approaches for work with *S. stercoralis* could also be beneficial. Applying the latest ‘omics technologies and long read sequencing to *Strongyloides* will allow full completion and annotation of *Strongyloides* genome assemblies, which will then underpin discovering the genomic and molecular basis of *Strongyloides* infection phenotypes, and facilitate reverse genetic approaches.

**Outstanding questions include:** can there be further improvement in using CRISPR-Cas9 and other reverse genetic methods with *Strongyloides;* is it possible to maintain the whole *Strongyloides* life cycle *in vitro*; how can the *Parastrongyloides* system be used to improve the study of *Strongyloides*?

## 3. Theme 2: human infection and disease

**Question 9. What are the different types of *Strongyloides* infections of people and animals?** (8 questions, electronic supplementary material, table S1)

While human *Strongyloides* infection is common, with estimates of 100–600 million people being infected worldwide [1,2,57,58], we know rather little of the nature of most of these infections, with studies instead focusing on people with complex and highly pathogenic infections that receive medical attention. Most people infected with *Strongyloides* likely have very low-intensity infections, which can be hard to diagnose (see **Question 10**). Further, disseminated infection (or hyperinfection) is also likely rare, or if it is not rare, then it is commonly undiagnosed. The duration of human infection is not well known, though it is thought to be chronic, but whether this is due to the lifespan of the parasitic females or due to autoinfection is unclear. Immunosuppression, particularly because of steroid treatment, can induce disseminated infection, as (less commonly) can severe malnutrition, alcoholism, immunodeficiency, and human T-lymphotropic virus type 1 infection [38,59], but it is unknown if there are other causes of dissemination, and so difficult to establish the multiple risk factors for disseminated infections (see **Question 6)**. Most human infection is with *S. stercoralis*, but in New Guinea there is also infection with *Strongyloides fuelleborni kellyi* [60] and in central Africa and Asia there are reports of zoonotic *S. fuelleborni* infection (e.g. [50,61]) (see **Question 7**).

**Outstanding questions include:** what is the range of types and duration of human *Strongyloides* infection in endemic populations; what are the causes and risk factors for disseminated infection, and for which species does this occur?

**Question 10. How can one best diagnose *Strongyloides* infection?** (5 questions, electronic supplementary material, table S1)

Compared with other soil-transmitted helminths (STHs; *Ascaris,* hookworm, *Trichuris*), *Strongyloides* is hard to diagnose, which has probably led to an underappreciation of its importance in endemic human populations. Faecal diagnosis using Baermann funnels or culture is the most sensitive, non-molecular diagnostic method, but is hard to do at large scale in epidemiological surveys. Overall, there is no agreed ‘gold standard’ diagnostic test available The development of improved molecular methods to diagnose and quantify *Strongyloides* and other STHs would be a very welcome development. If such methods were available then this could also be applied to understanding the distribution of *Strongyloides* infective stages in the wider environment, and so understanding the spatial and temporal infection risk (**Question 3**). Immunological diagnosis of infection is also possible, though with such methods there can be cross-reactions to other nematode infections; it can be difficult to clearly separate current and historical infection in some regions; antibodies may be maintained for up to 18 months after successful treatment; and cases of serologically negative individuals passing microscopically detectable *Strongyloides* larvae do occur [62].

**Outstanding questions include:** can a rapid, accurate, and easy-to-use diagnostic test be developed to diagnose infection at the population level and in clinical settings?

**Question 11. What is the best treatment for *Strongyloides* infection?** (6 questions, electronic supplementary material, table S1)

There are a range of anthelmintic drugs available that are used to treat STHs, including *Strongyloides*. The current US Centers for Disease Control and Prevention recommendation for treating *Strongyloides* infection is ivermectin, with albendazole as an alternative [63]. Commonly when people are infected with *Strongyloides* they will also be infected with other STHs, and in these settings a broad-spectrum anthelmintic is desirable; the same applies in veterinary settings. Human *Strongyloides* infections can be disseminated, and here therapy apart from anthelmintic treatment is required, but what is ideal and optimum is not well established [38], and given the relative rarity of disseminated infections it is difficult to see how controlled trials of different therapies can be established. As with all anti-parasitic drug treatment, drug resistance will evolve, with the only question being when and where. Within *Strongyloides* populations there is the possibility of natural variation in anthelmintic susceptibility, but drugs will also act as a selection pressure, changing populations' anthelmintic sensitivity and genetic diversity. Linking-back to the earlier consideration of *Strongyloides* species and populations (see **Question 7**), the population genetic structure of *Strongyloides* populations and the extent to which they interbreed will also affect the spread of resistance to anthelmintic drugs.

**Outstanding questions include:** what is the best treatment for uncomplicated and for disseminated *Strongyloides* infections; how does genetic variation in *Strongyloides* populations contribute to the evolution of drug resistance and its spread?

**Question 12. What are the life cycle and infection parameters that we need to know to better understand *Strongyloides* transmission and epidemiology?** (6 questions, electronic supplementary material, table S1)

Epidemiological science can model and predict patterns of infection in populations, and how those infections will respond to control measures that perturb the system. The underlying theory is now well established, though critical to applying this to make real-world predictions is having accurate parameters of aspects of infection. The challenges of estimating these parameters have been met for many STHs, but remain poorly studied for *Strongyloides*. Much of what needs to be known concerns within-host processes (see **Question 1**, **3** and **6**), which are challenging, though possible, to study in laboratory systems, but much harder in human infections.

**Outstanding questions include:** what is the effective fecundity of the parasitic generation (both daily rate and lifetime), and what is the effective fecundity of the free-living generation, and free-living adults’ contribution to that; what are the source, rate and magnitude of reinfection post-treatment?

**Question 13. How can we promote the importance and interest of *Strongyloides* within the context of it being one of the soil-transmitted helminthiases (a WHO Neglected Tropical Disease) and the One Health agenda?** (5 questions, electronic supplementary material, table S1)

*Strongyloides* as an STH is one of the WHO-defined Neglected Tropical Diseases, and arguably it is the most neglected of the STHs. This may be because it is rarer than other STHs and/or it is harder to diagnose, and so under-diagnosed (see **Question 10**). The source of human (and livestock) infection is intimately tied in with understanding what a *Strongyloides* species is and what host ranges *Strongyloides* species have (see **Question 7**), which is important to resolve to fully understand the source of human infection, and so how to control it.

The biological interest in *Strongyloides* and its life cycle has provoked two-thirds of the questions collected here, but answering these can be directly applied to understanding infection and disease in people and in livestock.

**The outstanding challenge** to all of us is to address and answer the questions we have posed ourselves, and to be advocates for the interest, importance and reward of studying the biology of *Strongyloides*.

## Data Availability

The data are provided in electronic supplementary material [64].
